# Identification of Metabolites in the Normal Ovary and Their Transformation in Primary and Metastatic Ovarian Cancer

**DOI:** 10.1371/journal.pone.0019963

**Published:** 2011-05-19

**Authors:** Miranda Y. Fong, Jonathan McDunn, Sham S. Kakar

**Affiliations:** 1 Department of Physiology and Biophysics, University of Louisville, Louisville, Kentucky, United States of America; 2 James Graham Brown Cancer Center, University of Louisville, Louisville, Kentucky, United States of America; 3 Metabolon, Inc., Durham, North Carolina, United States of America; University of Nebraska Medical Center, United States of America

## Abstract

In this study, we characterized the metabolome of the human ovary and identified metabolic alternations that coincide with primary epithelial ovarian cancer (EOC) and metastatic tumors resulting from primary ovarian cancer (MOC) using three analytical platforms: gas chromatography mass spectrometry (GC/MS) and liquid chromatography tandem mass spectrometry (LC/MS/MS) using buffer systems and instrument settings to catalog positive or negative ions. The human ovarian metabolome was found to contain 364 biochemicals and upon transformation of the ovary caused changes in energy utilization, altering metabolites associated with glycolysis and β-oxidation of fatty acids—such as carnitine (1.79 fold in EOC, *p*<0.001; 1.88 fold in MOC, *p*<0.001), acetylcarnitine (1.75 fold in EOC, *p*<0.001; 2.39 fold in MOC, *p*<0.001), and butyrylcarnitine (3.62 fold, *p*<0.0094 in EOC; 7.88 fold, *p*<0.001 in MOC). There were also significant changes in phenylalanine catabolism marked by increases in phenylpyruvate (4.21 fold; *p* = 0.0098) and phenyllactate (195.45 fold; *p*<0.0023) in EOC. Ovarian cancer also displayed an enhanced oxidative stress response as indicated by increases in 2-aminobutyrate in EOC (1.46 fold, *p* = 0.0316) and in MOC (2.25 fold, *p*<0.001) and several isoforms of tocopherols. We have also identified novel metabolites in the ovary, specifically N-acetylasparate and N-acetyl-aspartyl-glutamate, whose role in ovarian physiology has yet to be determined. These data enhance our understanding of the diverse biochemistry of the human ovary and demonstrate metabolic alterations upon transformation. Furthermore, metabolites with significant changes between groups provide insight into biochemical consequences of transformation and are candidate biomarkers of ovarian oncogenesis. Validation studies are warranted to determine whether these compounds have clinical utility in the diagnosis or clinical management of ovarian cancer patients.

## Introduction

Ovarian cancer is the most lethal malignancy of the female reproductive system and the 5^th^ cause of cancer death in women. It is estimated that 21,880 women will be diagnosed and 13,850 will die from this disease this year. The five-year survival rate at Stage I is 93.5% but drops to 27.6% at Stage IV, where a majority of cases are diagnosed due to a lack of symptoms at the earlier stages [1]. Current detection strategies include transvaginal ultrasound and blood CA-125 levels. However, both detection methods have shortcomings. With ultrasound, cancer could be mistaken for functional cysts in pre-menopausal women due to the dynamic nature of the ovarian surface [2]. CA-125 has a high false positive rate [2] that can arise from a variety of conditions including endometriosis, fibroids, hemorrhagic ovarian cysts, acute pelvic inflammatory disease, menstruation, first trimester pregnancy, and several other cancer types [3]. In addition, CA-125 is often not detectable in early stage ovarian cancer [4]. Alternative methods are being developed for patients who have normal CA-125 levels but are suspected to having recurrent disease based on clinical symptoms [5]. These methods include other potential biomarkers, the most promising being human epydidimus protein 4 (HE4), [6], [7], [8], [9], [10] despite detection rates of 50–60% in early stage ovarian cancer. A comprehensive study comparing the sensitivity of ovarian cancer biomarkers to discriminate between benign and malignant masses has been described [11] as well as the role of molecular markers in prognosis and therapy reviewed in [12]. It is important that suggested biomarkers have predictive value as indicated by sensitivity of 75% or greater as well as specificity of 99.6% to be able to detect early stage cancer when it is the most treatable [4].

One approach to identify disease biomarkers is to use information-rich analytical tools such as omics-scale biological methods to characterize the composition of the target tissue in health and disease. In this case, it is important to understand the biochemical alterations that are known to occur during neoplastic transformation. The first energy metabolism alteration in cancer cells was described by Otto Warburg, who showed cancer cells preference for glycolysis resulting in the generation of lactate for ATP production over the more efficient process of oxidative phosphorylation by the mitochondria [13]. This requires the cancer cells to increase their glucose uptake through the expression of several isoforms of glucose transporters (GLUT 1 to 9) [14] and to increase their glucose catabolism to compensate for the energy production loss, a fact that can be exploited in the clinical detection of neoplasm by positive emission tomography (PET) imaging [15].

The molecular mechanisms involved in the hyperactive glycolysis have been analyzed and some of key factors identified—including Akt, nuclear factor-κB (NF-κB), hypoxia-inducible factor-1 (HIF1), and p53 [14], [16], [17], [18], [19], [20]. The products of these genes are involved in cellular activation, nutrient import, and protection from apoptosis. These genes are known to interact in complex hierarchical webs. For example, HIF-1 can be modulated by other oncogenes such as Akt [14], K-Ras [21], and Her-2 [22] to increase the expression of several glycolytic enzymes. Other molecular mechanisms include transcriptional regulation by Myc to increase expression of transporters and glycolytic enzymes—in particular GLUT, hexokinase 2, and lactate dehydrogenase [14], [23]—as well as by the phosphoinositol-3-kinase (PI3K)/Akt/mammalian target of rapamycin (mTOR) pathway [24], [25], which is commonly overactive in carcinomas [26]. In addition, tumor metabolism differentially expresses glycoltyic isoenzymes, such as pyruvate kinase (PKM2), which can shift between a dimer and a tetramer to adapt to the energy requirements of the cells [27], [28]. However, PKM2 can also be by-passed by the accumulation of phosphoenolpyruvate (PEP) resulting in PEP-dependent phosphorylation and activation of phosphoglycerate mutase which produces pyruvate directly from 3-phosphoglycerate [29]. Vander Heiden *et al.* [29] hypothesized that this uncouples pyruvate production from ATP generation, maintaining an ATP/AMP ratio that does not inhibit glycolysis, and provides a significant pool of pyruvate as an anabolic precursor. Most of the research on tumor cell metabolism has focused on glucose utilization. When glucose is limited, solid tumors are forced to catabolize alternative substrates such as fatty acids, and amino acids as an alternative energy source.

However, oncogenesis can result in a diverse panel of metabolic alterations that could be tissue specific or generic across human cancers. Therefore, a comprehensive metabolic analysis of solid tumors could reveal valuable metabolites for both early diagnosis of cancer as well as to monitor disease progression and/or recurrence to inform clinical management of cancer patients. These biomarkers could conceivably be used as surrogate endpoints in clinical trials and could suggest new metabolic targets for cancer management as well as provide complementary targets for chemotherapy treatment. Metabolomics is a systematic analytical tool used for identification of biochemical metabolites from cellular processes, a term that includes several types of analyses ranging for nuclear magnetic resonance spectroscopy (NMR), mass spectrometry (MS), tracer-based studies, and metabolic footprinting [30]. While each of these methods has unique advantages, MS has established itself as the high-throughput and industrially stable approach to assess both the composition of diverse sample types as well as changes to that composition following perturbation. Although metabolomics has been around for decades, more recently it has garnered attention as a translational tool for the identification and treatment of cancer in the clinical setting, as well as for drug target development [31].

In a previous study, Denkert *et al.* [32] used gas chromatography MS/time-of flight (GC-MS/TOF) to compare borderline ovarian tumors to ovarian carcinomas. They identified 114 of 291 (39.1%) compounds and found an increased in proteinogenic amino acids, purines, pyrimides, and lipid membrane precursors in ovarian carcinomas vs. borderline tumors and interpreted these data to mean that carcinomas have higher cell proliferation rates. In addition to tumor metabolic analysis, urine samples from ovarian cancer patients have also been studied. Woo *et al.* [33] conducted a metabolomic-based study to find urinary biomarkers for ovarian and breast cancer using GC/MS. Two known biomarkers for breast cancer and 3 new biomarkers for ovarian cancer were identified: 1-methyladenosine, 3-methyluridine, and 4-androstene-3,17-dione. The ovarian cancer biomarkers were related to oxidative DNA damage and DNA methylation. Similarly, Slupsky *et al.* [34] collected urine samples from patients with early- and late-stage breast or ovarian cancer, as well as from healthy women, to obtain a metabolic profile using NMR. The concentration of specific metabolites decreased in patients with cancer, resulting in a unique profile. Alterations in intermediates of the tricarboxylic acid cycle (TCA) as well as molecules relating to energy metabolism and amino acids were observed.

Prior to this study, however, the metabolome of the normal ovary has not been studied, nor the changes that occur with neoplastic transformation and metastatic disease progression. In the present study, for the first time we report the metabolic profile of the normal human ovary and compare it to the metabolic profile of primary epithelial ovarian cancer (EOC) and metastatic tumors resulting from initial EOC (MOC) using GC/MS and LC/MS/MS.

## Results

### Identification of metabolites, statistical analysis, and pathway analysis

**Figure 1:**
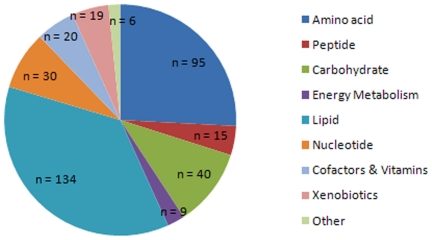
Class distribution of identified metabolites. n = number of metabolites in each class.

In samples from our three groups (normal, EOC, and MOC), 364 molecules were identified (Table S1) when compared to the Metabolon library containing 1,700 molecules. Identification was based on retention time, charge (*m/z*), preferred adducts, and fragmentation pattern of the molecule. The comprehensive library allowed for rapid identification with a high fidelity. These compounds included a large variety of classes, ranging from simple amino acids and peptides to carbohydrates, lipids, nucleotides, cofactors and vitamins, and xenobiotics (Fig. 1).

Data is a summation of individuals belonging to a group. Using one-way ANOVA with a Tukey post-test to identify differentially abundant metabolites across the three classes of tissue analyzed, 95 biochemicals were statistically significant and furthermore had a *p*≤0.05 in at least one of the pairwise comparisons (EOC vs. normal; MOC vs. normal; MOC vs. EOC). The identities of these metabolites are given in Table S2. Using the abundance profiles of these metabolites, supervised principal components analysis (PCA) was performed, yielding good separation of the three groups (Fig. S1).

Using Ingenuity Pathway Analysis (IPA), we identified the top 15 canonical pathways involved in EOC (Table S3) and MOC (Table S4). In almost all cases, these were related to amino acid metabolism and biosynthesis. Also of note, pyrimidine metabolism, purine metabolism, and glycoxylate and decarboxylate metabolism pathways only appeared in the case of MOC.

### Metabolic profile of the normal ovary and loss of function upon transformation

**Figure 2:**
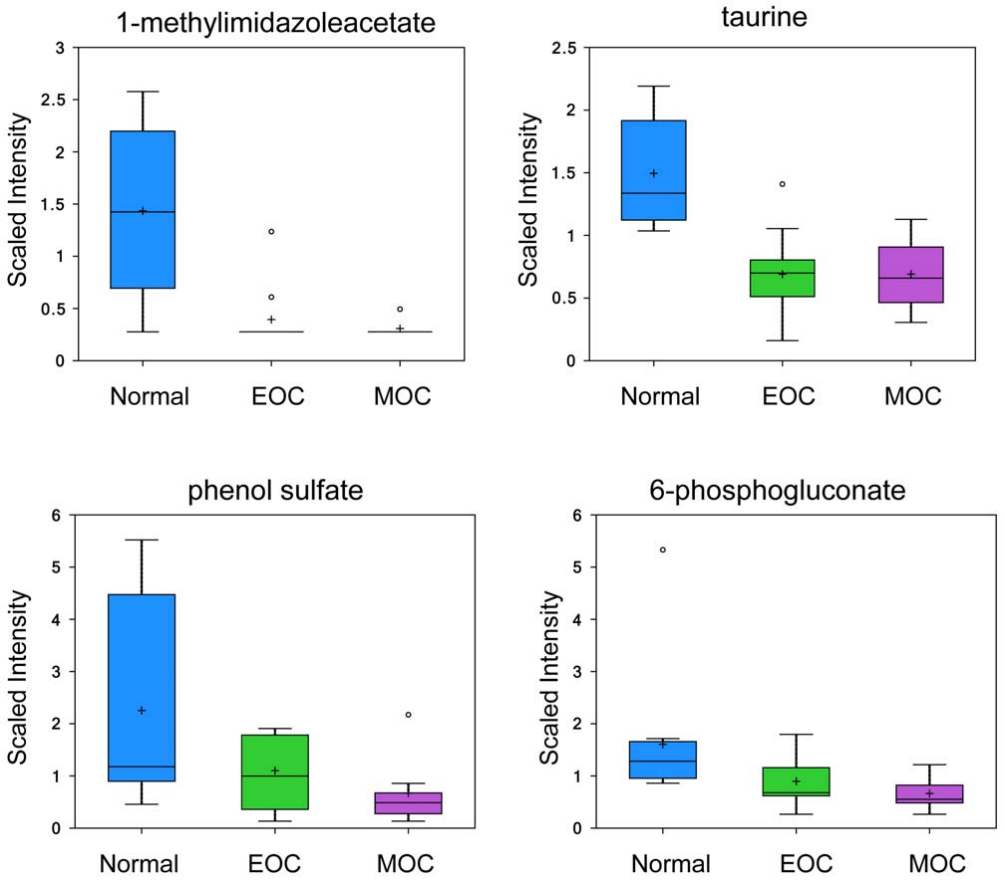
Significant metabolites present in the normal ovary and are reduced upon neoplastic transformation. 1-methylimidazoleacetate and taurine analyzed by LC/MS positive ion spray; phenol sulfate and 6-phosphogluconate analyzed by LC/MS negative ion spray. Box legend: + inside box represents mean value, bar inside box represents median value, upper bar represents maximum of distribution, lower bar represents minimum of distribution, circle represents extreme data points.

The first principal component separated the non-transformed ovarian samples from the transformed tissues (both EOC and MOC), while the second principal component identified a further set of biochemical alterations that corresponded with metastasis (Figure S1). Evaluation of the loadings plot further classified the compounds into loss/gain-of-function with either transformation or metastasis. Four metabolites were in high abundance in the ovary prior to neoplastic transformation: 1-methylimidazole acetate (−2.06 fold, *p*<0.001 in EOC; −2.18 fold, *p*<0.001 in MOC), taurine (−1.75 fold, *p*<0.001 in EOC, −1.97 fold, *p*<0.001 in MOC), phenol sulfate (−2.22 fold, *p* = 0.0535 in EOC; −3.0 fold, *p* = 0.0217 in MOC), and 6-phosphogluconate (−1.64 fold, *p* = 0.0538 in EOC, −1.92 fold, *p* = 0.0264; Fig. 2). These biochemicals have a significant drop in abundance upon transformation. Two of these metabolites (methylimidazoleacetate and 6-phosphogluconate) have previously been associated with normal ovarian function and the drop in their abundance can be considered a loss-of-function associated with transformation.

Methylimidazoleacetate is the main metabolite of histamine. This end product of histamine catabolism is formed by N-methylation in the imidazole ring to methylhistamine by histamine methyltransferase and a subsequent oxidative deamination in the side chain by type B monoamine oxidase. From studies it is known that as much as 70–80% of the histamine metabolized in the body is excreted in the urine as methylimidazoleacetate [35]. Thus, urinary methylimidazoleacetate being the major and specific histamine metabolite is a clear marker of any changes in histamine metabolism in the body. Ovarian histamine production occurs by tissue resident mast cells and has been shown to coordinate with ovulation [36].

Taurine is not involved in protein synthesis and/or has limited participation in biochemical pathways outside of peroxisomal formation of N-acyl lipid conjugates, such as bile acids and fatty acids. However, several functions have been demonstrated for taurine—such as osmoregulation, membrane stabilization, detoxification, antioxidation, modulation of ion flux, and as an inhibitory neurotransmitter or neuromodulator [37], [38], [39], [40], [41]. The roles of taurine in the reproductive system are multiple and complex. Taurine is the predominant amino acid in genital secretions—including seminal, uterine, and oviduct fluids [42], [43]. It has been demonstrated that the ovary contains the mRNA of a taurine transporter [44] and that the rat oviduct contains up to 10 µmol taurine/g tissue [45]. Taurine is also present in high concentrations in the rat and human uterus, and its concentration decreases with pregnancy [46], [47]. Despite all these data, the roles of taurine in the female reproductive system are largely unknown and there are no previous studies about its localization in these organs.

Phenol sulfate is a hepatically processed gut microfloral metabolite, and 6-phosphogluconate is an intermediate in the utilization of glucose within the pentose phosphate pathway, potentially signifying that glucose is restricted from entry into the pentose phosphate pathway in the healthy ovary and that upon transformation there is a higher affinity mechanism in place for this mechanism. This interpretation makes sense given that the pentose phosphate pathway produces both ribose for nucleotide biosynthesis, as well as two molar equivalents of NAD(P)H that could mitigate oxidative stress and aid in glutathione recycling.

Six compounds differentiated transformed ovarian tissue independent of whether the cancer was localized or metastatic (PC1>0; PC2 = 0). These compounds included several quaternary amines (betaine, carnitine, and erogthionine), the TCA cycle intermediates malate and fumarate and N-acetylglycine. Increased tissue quaternary amine concentrations are typically due to tissue demand for either choline or carnitine as the transporters for quaternary amines are selective but not specific [48]. In all, thirteen compounds containing quaternary amines were found to have increased tissue abundance in one or both of the ovarian cancer groups compared to the non-transformed ovarian tissue and two choline-containing lysolipids had significantly reduced abundance in the transformed ovarian tissue.

### Cancer cells have altered carbohydrate metabolism

**Figure 3:**
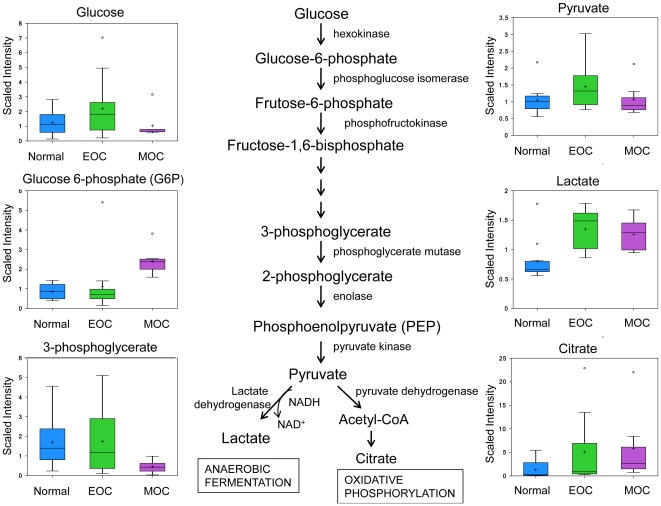
The glycolytic pathway converting glucose to pyruvate for anaerobic fermation to produce lactate or aerobic respiration of the citric acid for oxidative phosphorylation. Glucose, glucose-6-phosphate, fructose-6-phosphate, 3-phosphoglycerate, phosphoenolpyruvate (PEP), pyruvate, lactate, and citrate analyzed by GC/MS. Box legend: + inside box represents mean value, bar inside box represents median value, upper bar represents maximum of distribution, lower bar represents minimum of distribution, circle represents extreme data points.

One of the signature hallmarks of cancer is an altered glucose metabolism. In 1929, Otto Warburg first proposed that cancer cells utilized glucose differently than normal cells, preferring glucose for anaeroblic glycolysis instead of oxidative phosphorylation for the generation of ATP [13], resulting in increased lactate production and a lower pH than normal tissue, which in turn impairs DNA repair mechanisms [49]. Our results showed an increase in lactate in both EOC and MOC with a fold change of 1.46 (*p*<0.001) and 1.37 (*p* = 0.0076), respectively, when compared to normal ovarian tissue. Only MOC showed an increase in glucose-6-phosphate (2.91 fold, *p* = 0.0029). There were no significant changes in glucose, pyruvate, acetylphosphate, phosphate, pyrophosphate, or citrate between groups (Fig. 3). The increase in lactate coupled with no significant changes in citrate levels, indicate that glycolysis was not impeded but rather oxidative phosphorylation. Interestingly, another aspect of Warburg metabolism, hexose phosphate abundance, was only elevated in the MOC samples (data not shown).

The other carbohydrate with a significant increase in abundance in transformed ovarian tissue was fucose (2.75 fold, *p*<0.001 in EOC; 1.81 fold, *p* = 0.0103 in MOC). This finding may be due to a loss of function of specific ovarian glycosylation pathways since it has been demonstrated that normal ovarian tissue expresses a specific protein fucosylation pathway that results in the fucose moiety being directly coupled to the protein through Ser/Thr [50]. This ovary-specific glycosylation pathway is also unique in that the fucose is not the terminal sugar, but an internal sugar in the O-linked oligosaccharide.

### Increased fatty acid oxidation in EOC and MOC

**Figure 4:**
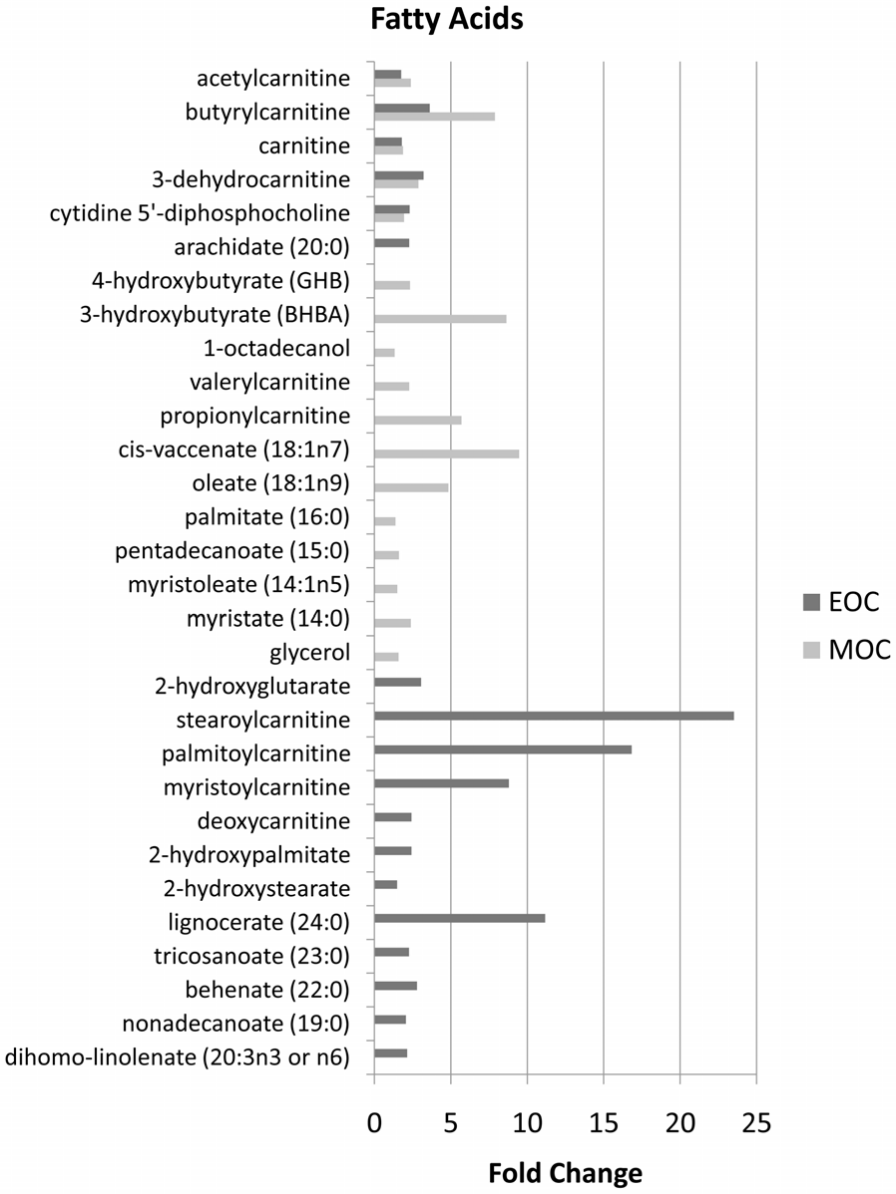
Upregulated fatty acids in EOC and/or MOC compared to normal ovarian tissue.

**Figure 5:**
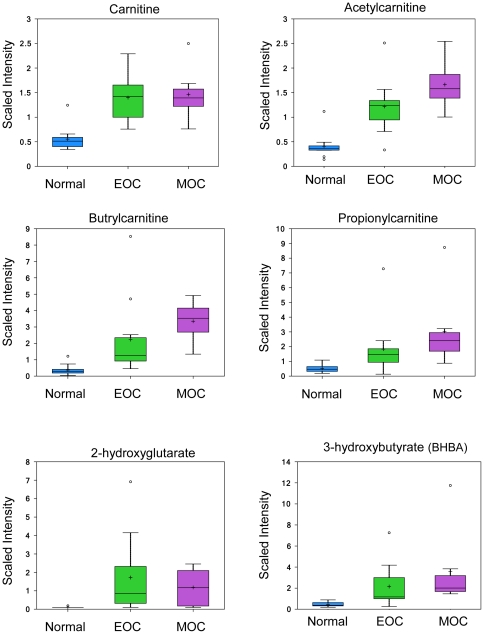
Carnitine and fatty acid metabolites. Carnitine, acetylcarnitine, butrylcarnitine, and propionylcarnitine analyzed by LC/MS positive ion spray. 2-hydroxyglutarate and 3-hydroxybutyrate analyzed by GC/MS. Box legend: + inside box represents mean value, bar inside box represents median value, upper bar represents maximum of distribution, lower bar represents minimum of distribution, circle represents extreme data points.

As an alternative to oxidative phosphorylation for ATP production, EOC and MOC prefer to utilize fatty acids as indicated by an increase in several fatty acids (Fig. 4) involved in fatty acid and carnitine metabolism—particularly carnitine (1.79 fold in EOC, *p*<0.001; 1.88 fold in MOC, *p*<0.001), acetylcarnitine (1.75 fold in EOC, *p*<0.001; 2.39 fold in MOC, *p*<0.001), butyrylcarnitine (3.62 fold, *p* = 0.0094 in EOC; 7.88 fold, *p*<0.001 in MOC), and propionylcarnitine increased 5.7 fold (*p* = 0.0047) in MOC only (Fig. 5). Carnitine has been recognized as a transport protein that delivers fatty acids into the mitochondria for β-oxidation. Endogenous acetylcarnitine has been used as an indicator of mitochondrial health through the balance of acetyl-CoA∶CoA by transferring the acetyl group to carnitine to form acetylcarnitine and thus provide acetyl groups for the synthesis of sterols, fatty acids, and ketone bodies [51].

The ketone body 3-hydroxybutyrate (BHBA) was upregulated 8.63 fold (p = 0.0056) in MOC compared to normal (Fig. 5). In addition, the cytosolic pool of acetyl-CoA is essential of de novo lipogenesis [52]. The excess production of cytoplasmic acetyl-coA compared to the mitochondrial capacity for its incorporation into the TCA cycle is demonstrated by the increased abundance of a panel of N-acetyl amino acids in the cancerous tissues—including N-acetylglutamate, N-acetylglycine, N-acetylthreonine, the neuroactive amino acids N-acetylaspartate (NAA) and N-acetyl-aspartylglutamate (NAAG), the polyamine degradation product N-acetylputrescine, and even N-acetylglucosamine-6-phosphate.

Interestingly, we also found that the recently described oncometabolite, 2-hydroxyglutarate had increased abundance in EOC (3.06 fold, *p* = 0.0114 in EOC; Fig. 5) [53].

### Enhanced phenylalanine catabolism

**Figure 6:**
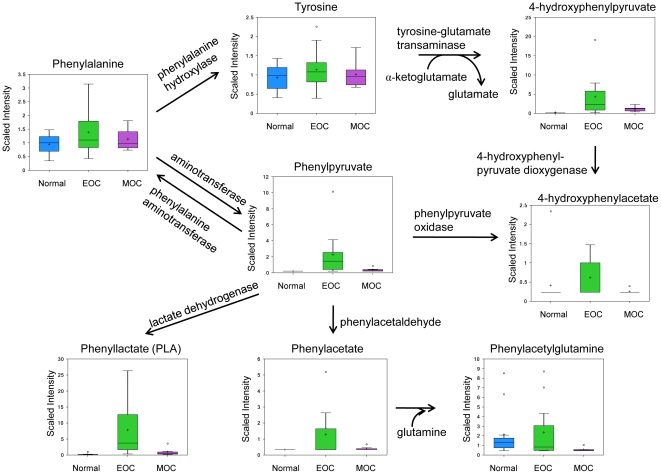
Phenylalanine metabolic pathway. Phenylalanine and tyrosine analyzed by LC/MS pos.; phenylpyruvate, phenyllacetate, phenylacetylglutamine, phenylactate, and 4-hydroxyphenylpyruvate via LC/MS negative ion spray; 4-hydroxyphenylacetate via GC/MS. Box legend: + inside box represents mean value, bar inside box represents median value, upper bar represents maximum of distribution, lower bar represents minimum of distribution, circle represents extreme data points.

Phenylalanine catabolism also results in the production of ketones, namely phenylpyruvate and 4-hydroxypyruvate. Major metabolites of phenylalanine catabolism were significantly increased in EOC compared to normal (Fig. 6). Phenylpyruvate increased 4.21 fold (*p* = 0.0098) in EOC only compared to normal but decreased −3.68 fold (*p* = 0.036) in MOC compared to EOC. Phenyllactate (PLA) increased 195.45 fold (*p*<0.0023) in EOC, a finding which is typically attributed to insufficient activity of phenylalanine hydroxylase [54]. Phenylacetate increased 1.93 fold (*p* = 0.0203) in EOC compared to normal only, whereas 4-hydroxyphenylpyruvate was increased 17.82 fold (*p* = 0.0069) in EOC compared to normal. Phenylalanine, tyrosine, phenylacetylglutamine, and 4-hydroxyphenylacetate were not significantly changed. Phenylalanine and its major metabolites—phenylpyruvate, PLA, and phenylacetate—induce oxidative stress in the hippocampus and cerebral cortrex via generation of reactive oxygen species, which was mitigated by α-tocopherol [55]. Phenylacetate has also been shown to have an inhibitory growth effect in ovarian cancer cell lines [56], whereas PLA can promote growth [57]. Therefore, it seems reasonable that ovarian cancer would favor the production of PLA over other alternative metabolites, consistent with our results. These metabolites are generated by transamination of phenylalanine and subsequent oxidation of the phenylpyruvate.

### Increased levels of tocopherols in MOC

**Figure 7:**
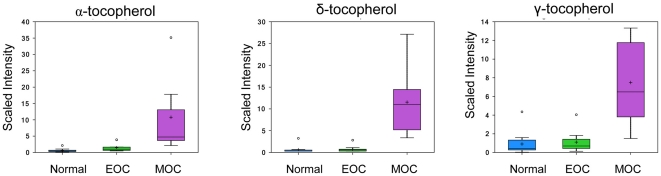
Three of the main tocopherol levels in normal, EOC, and MOC. Tocopherols analyzed via GC/MS. Box legend: + inside box represents mean value, bar inside box represents median value, upper bar represents maximum of distribution, lower bar represents minimum of distribution, circle represents extreme data points.

There are four main isoforms of tocopherols: α, β, δ, and γ, with α-tocopherol being the most biologically active form, accounting for approximately 90% of the Vitamin E found in animal tissues, where it serves as an antioxidant to quench free radicals and terminate lipid peroxidation [58], [59]. Hence it serves as an effective defense against radiation, which generates free radicals from water or biomolecules [60]. Metabolomic analysis showed a significant increase in α-, δ-, and γ-tocopherol levels in MOC. α-tocopherol increased 1,160.41 fold (*p*<0.001) compared to normal and 627.67 fold (*p* = 0.0023) compared to EOC. δ-tocopherol increased 1,775.51 fold (*p*<0.001) compared to normal and 1,950.08 fold (*p*<0.001) compared to EOC. γ-tocopherol increased 95.74 fold (*p*<0.001) compared to normal and 83.79 fold (*p*<0.001) compared to EOC (Fig. 7). As tocopherols are fat soluble, they are carried in the blood packaged in lipoproteins, mainly LDL and HDL, whereupon they are be transported to tissue and undergo uptake by the same mechanism by which lipids are delivered [58]. Uptake in the normal ovary is regulated by lipoprotein receptors [59]. Tocopherols have also been implicated in suppression of the immune system responsiveness by decreasing the reactive oxygen species and/or altering arachidonic acid metabolites [61]. Therefore, it seems reasonable that metastatic cancer would accumulate them to suppress the immune response and provide a defense against radiation treatment for cancer.

### Enhanced oxidative stress response

Ophthalmate is an analog of the reduced form of glutathione (GSH) with the thiol group of GSH replaced with a methyl group. Ophthalmate can be synthesized from 2-aminobutyrate and glutamate by the enzyme γ-glutamyl cysteine synthetase (GCS) to form γ-glutamyl-2-aminobutyrate [62], which can be catalyzed by glutathione synthetase (GS) to form ophthalmate [63] (Fig. 7). Ophthalmate has been indicated as a biomarker of oxidative stress as insufficient levels of GSH results in ophthalmate synthesis through activation of GCS. GSH is one of the most abundant intracellular antioxidants that protects the mitochondria from endogenous oxygen radicals [64] and also keeps enzymes and other cellular compounds in a reduced state [65], making it one of the most important cellular antioxidants, as its depletion leads to cell death. GSH has also been implicated in chemotherapy resistance through the activation of multi-drug resistant transporter 1 (MDR-1) [66], [67].

**Figure 8:**
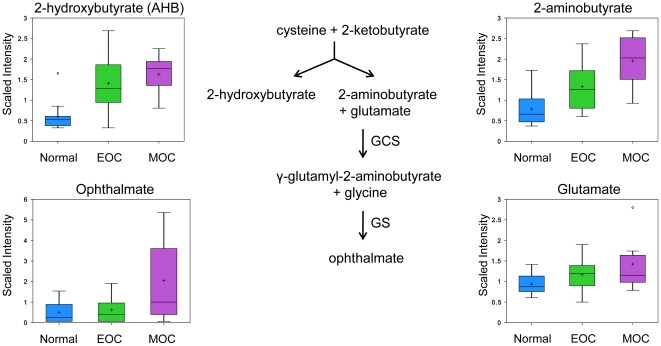
Ophthalmate biosynthesis pathway. 2-hydroxybutyrate and 2-aminobutyrate analyzed via GC/MS. Box plot of glutamate and ophthalmate analyzed by LC/MS positive ion spray. Box legend: + inside box represents mean value, bar inside box represents median value, upper bar represents maximum of distribution, lower bar represents minimum of distribution, circle represents extreme data points.

The oxidized form of glutathione (GSSG), GSH, γ-glutamyl-2-aminobutyrate, and ophthalmate can be detected in the serum in mice [67] so all have the potential for biomarkers. However, to date 2-aminobutyrate and ophthalmate have not been investigated in ovarian cancer. Here we report of the first time, significant increases of 2-aminobutyrate in both EOC (1.46 fold, *p* = 0.0316) and MOC (2.25 fold, *p*<0.001), suggesting an enhanced oxidative response. Ophthalmate increased 2.94 fold in MOC (*p* = 0.0128; Fig. 8). However, there were no significant changes in glutathione (reduced and oxidized states) or glutamate.

### Increased production of NAA and NAAG

**Figure 9:**
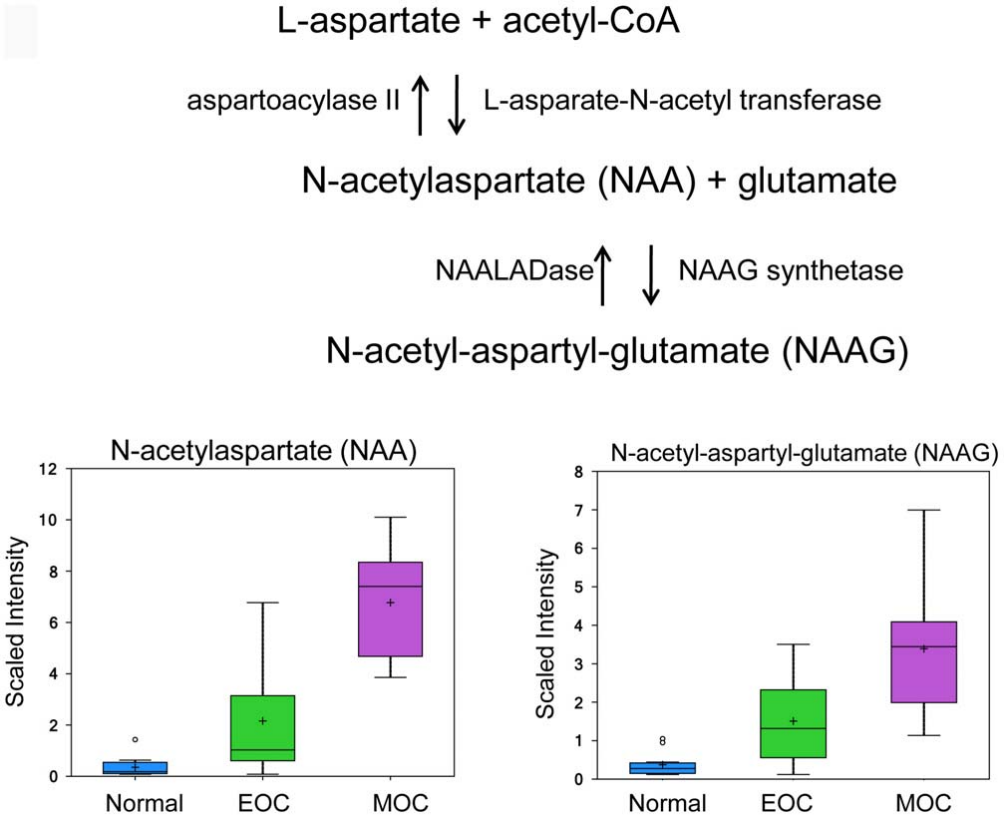
NAA and NAAG biosynthesis pathway. NAA and NAAG analyzed via LC/MS negative and LC/MS positive ion spray, respectively. Box legend: + inside box represents mean value, bar inside box represents median value, upper bar represents maximum of distribution, lower bar represents minimum of distribution, circle represents extreme data points.

N-acetylasparate (NAA) is a free amino acid found in the brain at very high concentrations that functions as an osmolyte in fluid balance to protect neurons against osmotic stress [68], [69]. It is also thought to serve as a source of acetate for lipid and myelin synthesis [70] and contribute glutamate for energy production in the neuronal mitochondria through a series of reactions [71], [72]. NAA is synthesized from L-asparate and acetyl-CoA by the enzyme L-asparate-N-acetyl transferase and hydrolyzed by the enzyme aspartoacylase II. NAA serves as a precursor for N-ascetyl-aspartyl-glutamate (NAAG) using the enzyme NAA synthetase (Fig. 8). Due to its packaging with glutamate, the physiological role for NAAG has been difficult to identify, however, it fulfills the criteria of a neurotransmitter as it is packaged into synaptic vesicles and released in a Ca^2+^-dependent manner from nerve terminals [73]. It has been proposed to serve as a shuttle for glutamate to activate the glutamate receptor mGluR3 due to the cytotoxic nature of glutamate [74], [75]. More recently, it has been used to diagnose brain disorders. Moreover, NAA concentrations have been found in a patient with ovarian mucinous cystadenoma [76] and in ovarian cyst fluid of serous ovarian tumors [77], although a physiological role in the ovary has not been determined. Here we report that NAA and NAAG, two free amino acids, were detected in the normal ovary with significantly increased levels in EOC showing a fold change of 3.50 (*p* = 0.0301) and 2.19 (*p* = 0.0352), respectively, with further increases in MOC with a fold change of 85.60 fold (*p*<0.001) and 8.09 (*p*<0.001), respectively (Fig. 9).

NAAG is broken down by N-acetylated alpha-linked acidic dipeptidase (NAALADase), a NAAG-specific catabolic enzyme [78]. NAALADase is composed of three family members: NAALADase I, NAALADase L, and NAALADase II. NAALADase II has been found to be highly expressed in the ovary when identified by Northern blot and reverse-transcription PCR [79].

## Discussion

Metabolomics has been widely used to identify biomarkers for various disease states—including diabetes [80] and atherosclerosis [81]—using blood, urine, cells, and tissue. Of import, metabolomics has provided a comprehensive technique to identify biomarkers for cancer—including breast [82], [83], [84], [85], ovarian [32], [33], [86], prostate [87], [88], colorectal [89], [90], [91], and gastric cancer [92]. Identification of biomarkers is of the utmost importance as it can help diagnose diseases at an earlier stage, leading to a better prognostic outcome, when used in conjunction with existing methods, such as transvaginal ultrasound in the case of ovarian cancer.

In this study, the comprehensive metabolic profile of normal ovaries, EOC, and MOC were compared using GC/MS or LC/MS/MS. Significant changes in energy utilization were detected as well as an enhanced oxidative stress response. Similar to the study by Denkert *et al.* [32], we have found increased levels of amino acids in EOC and MOC vs. normal. However, contrary to their study, the lysolipid levels were either not significantly changed or downregulated in EOC and/or MOC. Woo *et al.* [33] found urinary biomarkers for ovarian cancer using GC/MS related to DNA oxidative damage and DNA methylation: 1-methyladenosine, 3-methyluridine, and 4-androstene-3,17-dione. We found no significant changes in N1-methyladenosine or 4-androstene-beta,17beta diol disulfate based on tissue samples, perhaps due to the high amount of GSSG found in our samples coupled with a reduced amount of GSH, protecting the tissue from oxidative damage or from a more efficient excretion of the metabolites in these patients. We also found increased tocopherols in MOC, which are best known for their antioxidant properties. More recently, urine metabolite profiling in breast and ovarian cancer showed that metabolite concentrations correlated with both cancers compared to healthy individuals [34]. Consistent with the aforementioned study, Odunsi *et al.* [86] were able to separate sera from healthy individuals from that of EOC patients using (1)H-NMR spectroscopy. Applying unsupervised PCA analysis as well as supervised Soft Independent Modeling of Class Analogy (SIMCA) allowed for pattern recognition. They were able to correctly identify increases in alanine, valine, glucose, and 3-hydroxybutyrate (a ketone body) in EOC sera. In our samples, we showed an increase of 3-hydroxybutyrate in MOC with a borderline significant increase in EOC. However, the other metabolites were not significantly altered.

Additional metabolic analysis in colorectal tissue [89], [91] and in gastric cancer metastases [92] samples showed increases in glycolysis shown by the increase in lactate and fatty acid metabolism, and decreases in TCA intermediates. Lactate and phenylalanine have yielded satisfactory sensitivity and accuracy in differentiating oral squamous cell carcinoma from oral leukoplakia [93]; however, these metabolites could be generic across multiple cancers.

One of the proposed biomarkers for tumor progression and invasiveness in prostate cancer is sarcosine, also known as N-methylglycine [94], although there is some debate whether sarcosine can be detected in the serum [95] or urine [96]. Sarcosine can be synthesized from glycine by the enzyme glycine N-methylransferase or from dimethylglycine by the enzyme dimethylglycine dehydrogenase, which in turn is demethylated from betaine. In our samples, we found a significant increase in betaine in both EOC and MOC compared to normal. However, dimethylglycine and sarcosine were not significantly different, possibly due to a high biological variability between samples, which could be clarified with an increased sample size.

Based on the data we have collected, we have identified possible candidates for biomarker analysis for both preliminary cancer diagnosis and metastatic disease progression. The dramatic increase of tocopherols in MOC makes these molecules attractive candidates for aggressiveness and/or progression. Further investigation is needed to determine the potential utility of these initial findings.

## Materials and Methods

### Histopathology

For metabolic profiling, 30 patient tissues were obtained from University of Alabama, Birmingham, Comprehensive Cancer Center and stored at −80°C. Type and stage was determined by evaluation by a pathologist. Tissues included 12 normal ovarian samples (mean age 48.6±7.6 years), 11 primary ovarian adenocarcinomas ranging from Stage I through Stage IIIC (mean age 56.5±9.8 years), and 7 metastatic tumors in the omentum resulting from initial ovarian adenocarcinomas ranging from Stage IIIC through Stage IV (mean age 62±8.2 years). Patients with previous diseases and treatments were excluded from the study.

### Metabolic Profiling

100 µg of frozen biopsy tissue was submitted to Metabolon, Inc. (Durham, NC) for sample extraction and analysis. In brief, Metabolon performed cold methanol extraction of mechanically disaggregated tissue samples and these extracts were split into three aliquots. The reproducibility of the extraction protocol was assessed by the recovery of xenobiotic compounds spiked into every tissue sample prior to extraction. These aliquots were processed and characterized by one of the three analytical methods previously described: UHPLC-ESI-MS/MS in the positive ion mode, UHPLC-ESI-MS/MS in the negative ion mode and sialylation followed by GC-EI-MS. Chromatographic timelines were standardized using a series of xenobiotics that elute at specified intervals throughout each chromatographic run. The technical variability of each analytical platform was assessed by repeated characterization of a pooled standard that contained an aliquot of each sample within the study. The platform for sample analysis has been described in detail [97], [98]. However, the combination of the Metabolon platform with ovarian tissue is novel.

## Supporting Information

Figure S1 Supervised PCA separated normal ovarian tissue from ovarian cancer (PC1; blue→green and purple) and localized tumor from metastasis (PC2; green→purple).
(TIF)

Table S1 List of metabolites identified through mass spectrometry.
(XLS)

Table S2 ANOVA analysis of metabolites grouped by class of compound.
(XLS)

Table S3 Ingenuity pathway analysis for the top 15 canonical pathways for EOC.
(XLS)

Table S4 Ingenuity pathway analysis for the top 15 canonical pathways for MOC.
(XLS)
