# Supplementary figures and images for: Identification of Metabolites in the Normal Ovary and Their Transformation in Primary and Metastatic Ovarian Cancer
Source: PLoS One. 2011 May 19;6(5):e19963. doi: 10.1371/journal.pone.0019963 (PMC3098284; doi:10.1371/journal.pone.0019963)

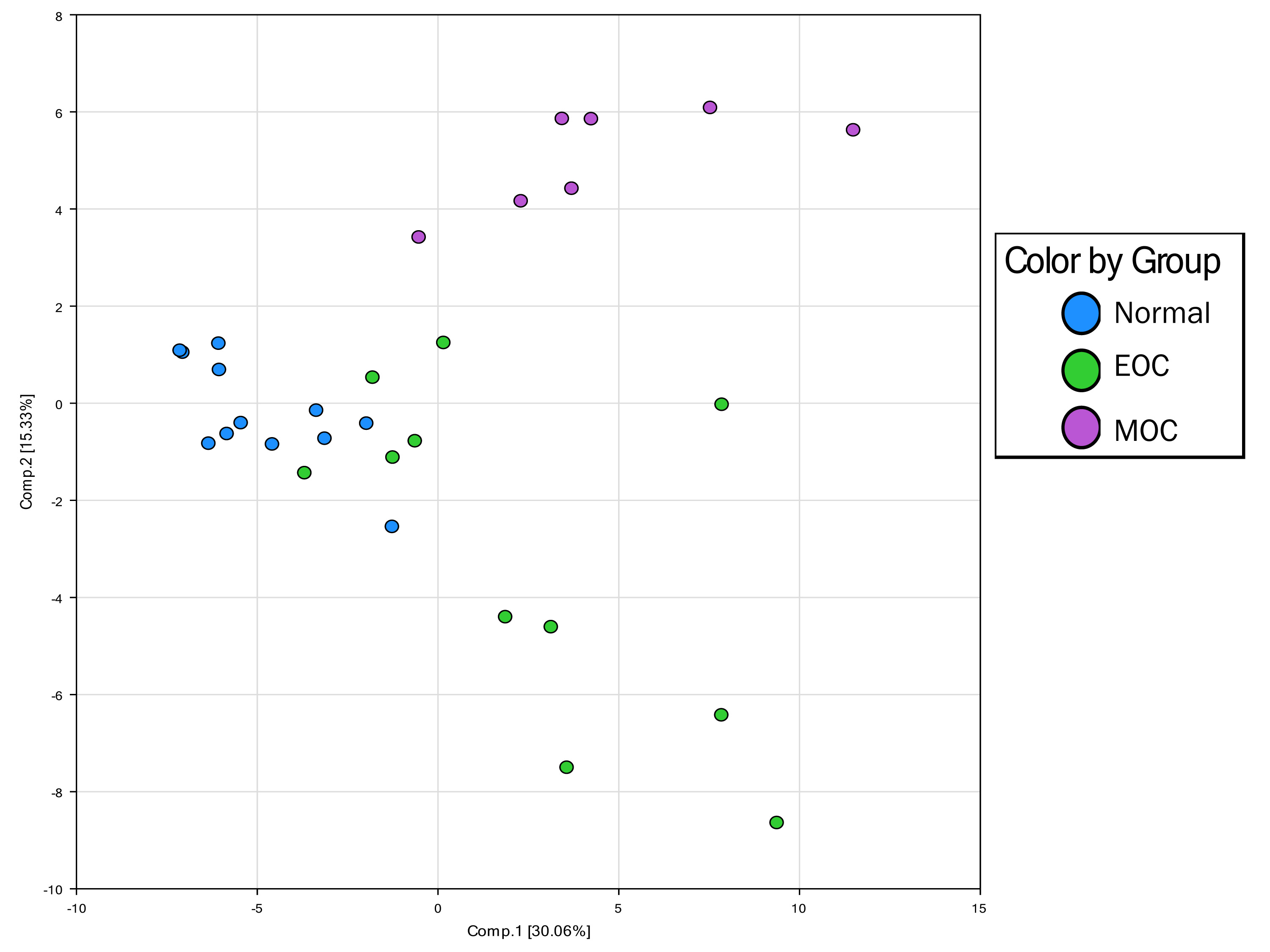

Supplement: Figure S1 — Supervised PCA separated normal ovarian tissue from ovarian cancer (PC1; blue→green and purple) and localized tumor from metastasis (PC2; green→purple). (TIF) [file pone.0019963.s001.tif]
